# Barriers and facilitators to the uptake of an antimicrobial stewardship program in primary care: A qualitative study

**DOI:** 10.1371/journal.pone.0223822

**Published:** 2020-03-05

**Authors:** Lianne Jeffs, Warren McIsaac, Michelle Zahradnik, Arrani Senthinathan, Linda Dresser, Mark McIntyre, David Tannenbaum, Chaim Bell, Andrew Morris

**Affiliations:** 1 Lunenfeld-Tanenbaum Research Institute, Sinai Health System, Toronto, Ontario, Canada; 2 Keenan Research Centre, Li Ka Shing Knowledge Institute, St Michaels Hospital, Toronto, Ontario, Canada; 3 Ray D. Wolfe Department of Family and Community Medicine, Sinai Health System, Toronto Canada; 4 Department of Family and Community Medicine, University of Toronto, Toronto, Canada; 5 St Michaels Hospital, Toronto, Canada; 6 Antimicrobial Stewardship Program, Sinai Health System and University Health Network, Toronto, Canada; 7 Department of Medicine, Sinai Health System, University Health Network, and University of Toronto, Toronto, Canada; Imperial College London, UNITED KINGDOM

## Abstract

The overuse of antimicrobials in primary care can be linked to an increased risk of antimicrobial-resistant bacteria for individual patients. Although there are promising signs of the benefits associated with Antimicrobial Stewardship Programs (ASPs) in hospitals and long-term care settings, there is limited knowledge in primary care settings and how to implement ASPs in these settings is unclear. In this context, a qualitative study was undertaken to explore the perceptions of primary care prescribers of the usefulness, feasibility, and experiences associated with the implementation of a pilot community-focused ASP intervention in three primary care clinics. Qualitative interviews were conducted with primary care clinicians, including local ASP champions, prescribers, and other primary health care team members, while they participated in an ASP initiative within one of three primary care clinics. An iterative conventional content analyses approach was used to analyze the transcribed interviews. Themes emerged around the key enablers and barriers associated with ASP implementation. Study findings point to key insights relevant to the scalability of community ASP activities with primary care providers.

## Introduction

Antimicrobial resistance (AMR) is a complex global public health threat, [1,2] and is strongly associated with antimicrobial consumption. [3,4] It is estimated that three-quarters of antimicrobial use occurs in the community, where primary care practitioners account for the bulk of antimicrobial prescribing. [1,5,6] These outpatient antimicrobial prescriptions are most often issued for upper and lower respiratory conditions including sinusitis, otitis media, pharyngitis, acute bronchitis and pneumonia. [5–7] However, up to 50% of these prescriptions may be unnecessary or inappropriate. [8,9] The overuse of antimicrobials in primary care can also be linked to an increased risk of antimicrobial-resistant bacteria for individual patients. [10] Together these factors indicate the importance of addressing unnecessary antimicrobial prescribing in primary care in order to limit AMR. [11]

Most efforts to optimize antimicrobial use have focused on inpatient settings. [12,13] Hospital–based antimicrobial stewardship programs (ASP) have been developed with the aims of limiting antimicrobial therapy to those infections likely to benefit from such treatments, shortening the duration of therapy, and reducing the use of broad-spectrum antimicrobials to reduce collateral damage, including infections with *C*.*difficile*. [13] ASPs are multi-faceted and use interventions to change prescribing behaviour that may include education, de-escalation, clinical decision support, formulary restriction, and audit and feedback. [13,14]

The development of comprehensive multi-faceted ASPs for primary care settings is less studied, and how best to implement ASP programs in these settings is still unclear. [15–17] Some individual interventions have demonstrated effectiveness in reducing antimicrobial use in primary care, including communication skills training, education interventions, point-of-care testing, electronic decision support systems, and delayed prescribing. [18–20] However, there are few studies of the views of community providers regarding the usefulness and feasibility of these interventions when delivered as part of a multi-faceted program. Most studies have focused on providers’ views of one or a few interventions. [21, 22]

For example, French general practitioners viewed an intervention of a dedicated prescription for antimicrobials as restrictive and excessive.[22] Understanding how primary care providers view proposed multifaceted programs may inform which aspects are acceptable or feasible in the primary care setting, and thus how likely they are of being adopted. In this context, we conducted a qualitative study to evaluate perceptions of primary care prescribers of the usefulness and feasibility, and experiences associated with the implementation of a pilot community-focused ASP intervention in three primary care clinics.

## Methods

### Study setting

The Primary Care Antimicrobial Stewardship Program (PC-ASP) was implemented within three primary care Family Health Teams (FHTs) between July 2016 and April 2017 in Toronto, Canada. In Canada, health care is publicly funded and primary medical care is provided by general practitioners and nurse practitioners to persons of all ages. The participating FHTs are interprofessional urban primary care clinics in Toronto, Canada that provide primary care to more than 30,000 patients. Two clinics are affiliated with academic teaching hospitals, with a primary role in the training of family medicine residents.

### Study design

Qualitative interviews were conducted with primary care clinicians, including local ASP champions, prescribers, and other primary health care team members, while they participated in an ASP initiative. Ethics approval was received from the Sinai Health System Research Ethics Board; University Health Network Research Ethics Board and St. Michaels Hospital Research Ethics Board.

### Study participants

All physician providers approached to participate in this study were university-appointed members of the same Department of Family and Community Medicine at the University of Toronto, Canada. Nurse practitioners employed in these clinics were also approached, as they independently prescribe antimicrobials in these settings. One site (Site 3) did not have a pharmacist or nurse practitioner. While characteristics of those who declined to participate were not collected, both they and study participants were similarly involved in the teaching and training of family medicine resistant trainees, as well as the provision of clinical care to person attending these clinics.

### Intervention description

The PC-ASP was adapted from the Sinai Health System-University Health Network hospital-based Antimicrobial Stewardship Program in Toronto, Canada, based on the input of community providers regarding the feasibility of various hospital ASP elements. [23] Additional interventions shown to be effective in primary care settings were incorporated. [15,18, 24] This pilot PC-ASP was limited to adults with four types of infections (sore throat, sinusitis, bronchitis and cystitis). It included a multi-faceted knowledge translation (KT) approach, which is acknowledged as resulting in more effective knowledge translation than single interventions. [18] The overall objective was to develop a model program that was feasible and would be scalable across different primary care settings. A brief description of each individual element of the multi-faceted intervention is included as Table 1.

**Table 1:** A description of each element of the multi-faceted PC-ASP intervention.

Individual Intervention	Brief Description
Local Site Champions [25]	• Individuals working at a site, known to clinic staff, knowledgeable of local site logistics, and willing to champion ASP efforts at their site. (administrative staff, pharmacist, 1–2 physicians or nurse practitioner depending on the site)
Clinic Work Flow Analyses [26]	• Identifying stewardship opportunities in the process of patient flow through each clinic from requesting an appointment, the various staff encountered, their roles in encounters suspected of being an infection, and different places in the clinic (reception, office assistant or nursing station, clinical exam room) the patient moved through
Audit and Feedback Reports [27,28]	• Clinic records were audited every 3 months to abstract antimicrobial prescribing information for every respiratory and urinary tract infection (women only) identified. 1-page reports detailed overall prescribing rates compared to the other clinics, and antimicrobial choices for the four targeted infections
Prescriber-focused E-learning modules [29]	• A 2 hour set of 5 modules (introduction to antimicrobial resistance and stewardship, and 1 for each of the 4 infections) explaining the infection-specific use of the decision aids, key communication points and patient education aids
Prescribing Clinical Decision Aids [30–32]	• 1-page decision support aids for prescribing decisions, or guideline summaries, providing infection-specific diagnostic criteria, indications for antimicrobials, recommended antimicrobials, use of delayed prescriptions, supportive care, and symptoms suggesting serious complications
Communication Scripts [33,34] for Patient Engagement	• Education about antimicrobial-specific communication skills, key stewardship points to communicate to patient specific to each condition, and sample scripts of ways to communicate points
Delayed Antimicrobial Prescriptions [35]	• Suggested situations and instructions to patients for delaying a prescribed antimicrobial
Safety Netting Messages [36]	• Specific follow up advice to patients where antimicrobials were not prescribed, to ensure any worsening infections received medical care
Environmental Messaging, Patient Education [15,19]	• Patient handouts reinforcing stewardship rationale, adverse antimicrobial effects, supportive care and safety netting. Waiting room posters, videos of stewardship information were used in some clinics

## Recruitment and data collection methods

The names of potential participants at one site were obtained from the Principal Investigator (WM), who was also a practitioner at the site, and from the recruited local champions at the other two sites. All family physicians, nurse practitioners and pharmacists were approached with an email invitation. An email recruitment letter was sent by the Study Coordinator to invite participants to participate in an interview. Informed consent was obtained prior to the interviews. The interviews occurred during the implementation of the PC-ASP at each site. An interview guide was developed to evaluate participants’ perceptions and experiences associated with their involvement in the PC-ASP.

Key questions included: What factors do you think influence the prescribing practices for the following conditions in your FHT clinic: 1) sore throat, 2) sinusitis, 3) bronchitis, and 4) cystitis? What were the supports and enablers related to your participation in the Primary Care Antimicrobial Stewardship Program? Looking forward, what resources need to be in place to support continuity of the Primary Care Antimicrobial Stewardship Program? What recommendations do you have to improve the Primary Care Antimicrobial Stewardship Program: what would you change, what would you add?

An experienced research coordinator in qualitative methods and experience with ASP in hospitals conducted all interviews. The research coordinator had no pre-existing relationships with interview subjects. Interviews were conducted both in person and by telephone depending on the preference of the study participant. All interviews were audio recorded and transcribed verbatim for analysis.

### Data analysis

An iterative conventional content analyses approach was used to analyze the transcribed interviews. [37,38] This analytical process involved two researchers (LJ and MZ) reviewing the transcripts line-by-line separately to identify sections of text that served as codes. The Principal Investigator (LJ) and the research coordinator (MZ) conducted the main analysis of the interviews to develop the initial coding schema. Neither had any relationships with the study participants at the 3 sites. During the second phase, the researchers met to determine, through consensus, the codes and categories and develop the initial coding schema. Over a series of analytical meetings, the coding schema was refined, with the final step including a review of the emergent coding schema by one of the principal investigators (PI) [LJ] and all the transcripts to ensure categorical data was included in the final coding schema.[37,38] The research coordinator met with the two PIs (LJ and WM) as the interviews were being conducted and the coding schema developed and over the course of the interviews would probe participants around emerging themes.

### Sample characteristics

All providers who prescribed antimicrobials in these clinics as well as site pharmacists were eligible to participate. In total, 23 interviews were conducted as saturation was achieved within this dataset with no new insights emerging from the interviews.

The HCPs interviewed included: 19 family physicians, 3 pharmacists, and 1 nurse practitioner (NP). There were 9 providers from Site 1 (out of 13), 7 from Site 2 (out of 25) and 7 from Site 3 (out of 9). Site 2 is a larger clinic with a number of part-time providers that varies each year. The average length of interviews was 16.5 minutes with a range of 11–32 minutes.

## Findings

The narrative dataset yielded a number of themes that could be considered broadly as those facilitating involvement in ASP activities in the primary care setting, and factors that were seen as barriers to optimal antimicrobial prescribing.

### Facilitators to the primary care antimicrobial stewardship program

Providers identified the following factors they saw facilitating ASP activities in their clinic: 1) being engaged by local champions; 2) having flexible and relevant learning strategies; 3) accessing information, resources and reminders; 4) valuing the knowledge of prescribing performance through the audit and feedback reports; and 5) creating heightened awareness about antimicrobial resistance and stewardship.

#### 1. Being engaged by local champions

This theme reflected how local champions were perceived by study participants in influencing them to participate in the PC-ASP. The majority of participants described active involvement of their local champion(s); a few described their local champion to be more “hands-off”. Key roles played by the local champion(s) included increasing awareness of the PC-ASP by email correspondence regarding the launch of the PC-ASP and facilitating educational sessions (e.g., lunch and learns, team meetings) to FHT members. Other roles included: providing ongoing updates on the PC-ASP; sharing audit and feedback results to FHT members of antimicrobial prescribing practices; and reminding FHT providers to complete the educational modules and access resources available to them (e.g., decision aids, templates).

“*Our local champion (team pharmacist) was helpful in supporting it and getting this information out*. *I saw her as the leader and everything flowing in through her*. *There were fairly regular emails updating us about the status of the program*.*”* (Site 1 Family Physician 1)

“*They (pharmacist and staff physician) are our site champions [and] they are centrally involved with the project*. *We receive feedback on how we are doing overall*, *and I think it is always nice to have comparators to other FHTs*.*”* (Site 2 Family Physician 2)

“*They sent out an email*, *they held that meeting*. *They put the handouts in every room*, *they put the decision aids on the walls in every room and then they send follow-up emails like this is the percentage of how the clinic is doing—graphs that they send out*.*”* (Site 3 Family Physician 1)

#### 2. Having flexible and relevant learning strategies

Participants emphasized that flexible learning and multi-modal approaches were crucial for the uptake and future scalability of the ASP to other primary care sites. Some participants preferred a self-directed on-line option, while others preferred going through the learning modules in a group session where they could discuss more. In one site, the local champion uploaded the learning modules directly onto the desktop computers used by the providers. Although many participants described the learning modules as repetitive and redundant, they valued the content on each of the four conditions and viewed it as relevant. However, a subset of participants reported that they did not access to or use the learning modules.

“*The approach to AS in any context needs to be multimodal*, *very flexible in approach as there are many other people who learn differently and have different learning needs*.*”* (Site 2 Family Physician 7)

“*They [e learning modules] were relevant and useful*. *There was a little bit of pushback*. *I think people liked the idea of the project and the rationale behind it*. *I know based on the email we got that I think only half of the group did the modules*. *I did get some informal feedback about you know that they had done the modules just not said that they did it or submitted for the credit*.*”* (Site 3 Family Physician 1)

#### 3. Accessing information, resources and reminders

Having easy access to antimicrobial stewardship information, resources, and reminders on the computers was noted as influencing its use in daily practice the most. One site created a screensaver and icon that linked directly to the PC-ASP decision aids and patient handouts. The initial flagging of patients by other clinical staff (e.g., secretary or clinical assistant) and uploading of a decision-support template also triggered prescribers amidst competing demands and busy workloads to enact the PC-ASP components into practice. Study participants identified the value of having handouts for patients and having incentives, such as continuing medical education credit for participation.

“*We have ongoing reminders*, *emails every week and team meetings to review how we are doing*. *There are screensavers on our computers*, *posters up everywhere*. *The patient handouts are part of the template*, *but they are separate they are on the desktop*. *There are also training modules*, *self-learning that you went through*.*”* (Site 1 Nurse Practitioner)

“*The decision aids in the rooms were very helpful I used those*, *and I continue to use them*. *Someone physically put the decision aids in our room that was very helpful*.*”* (Site 3 Family Physician 6)

#### 4. Valuing the knowledge of prescribing performance through audit and feedback reports

The majority of participants valued the audit and feedback component of the PC-ASP and comparison of prescribing practices across the three sites, describing it as *“helpful”*, *“well presented”*, *“made you stop and think for a second”*, and *“reinforce not to overuse antibiotics”*. This is illustrated in the narrative excerpts below.

“*That [audit and feedback] was very helpful*. *It was quick and easy to read and only took a minute or a couple minutes to review and get the points across*. *It was very well presented*. *Keep that feedback audit loop going would have the most impact ongoing throughout my practice*.*”* (Site 1 Family Physician 1)

“*We love data that tells us how we are performing so those are really helpful*. *I liked the way that there was a root cause analysis of what the various reasons for prescribing*. *I think it was very nicely laid out*, *easy to see [and] scan*.*”* (Site 2 Family Physician 7)

Study participants also provided recommendations around the audit and feedback reports. These included providing audit and feedback results to the individual prescriber, having data from other prescribers (data from residents and nurse practitioners were excluded in this study), and conducting ongoing audits with benchmarking of prescribing practices.

“*Monitoring and feedback with a set of benchmarks and targets*. *If we can find ways to provide audit and feedback information that is great*, *that would be really helpful it then keeps the attention of the individuals*.*”* (Site 1 Family Physician 9)

“*Audit and feedback is also useful because it motivates you to do better or to see what changes you have made and there are some discussion whether individual audit and feedback or group audit and feedback would be more useful*. *Ideally that would be something very interesting to have access to*.*”* (Site 3 Family Physician 4)

#### 5. Creating a heightened awareness about antimicrobial resistance and stewardship

Study participants described how the ASP heightened their awareness of their antimicrobial prescribing practices for the four conditions. Some study participants also described changing their antimicrobial prescribing practices based on the knowledge gained and resources provided in the PC-ASP. These changes included being more systematic in diagnostic approach, delaying prescriptions, shortening the duration of antimicrobial therapy, and selecting different antimicrobials to treat infections. The following quotes provide examples of this theme.

“*I think overall awareness and presenting the evidence*. *I think awareness and sort of a better understanding of diagnostic criteria and decision points around how to decide whether they need antibiotics*.*”* (Site 1 Family Physician 4)

“*I think the various initiatives have made me think more about shortening the duration of treatment*, *about giving a delayed prescription*.*”* (Site 1 Family Physician 7)

“*It has guided me in terms of antibiotic selection to use more the first-line instead of the second or third line*.*”* (Site 3 Family Physician 2)

### Barriers to antimicrobial stewardship within a primary care setting

This theme captured factors providers believed affected their ability to prescribe antimicrobials optimally. These included 1) feeling pressured by patients to prescribe antimicrobials; 2) being influenced by situational factors affecting prescribing decisions; 3) experiencing diagnostic uncertainty based on clinical factors; 4) believing that providers were already prescribing judiciously with little room for improvement; and 5) having time pressures affecting prescribing and learning about antimicrobial stewardship.

#### 1. Feeling pressured by patients to prescribe antimicrobials

Prescribers universally reported feeling pressured by patients to prescribe antimicrobials. Several participants shared that a patient would request an antimicrobial based on their preconceived notion and previous experience of feeling better after being prescribed antimicrobials. Participants also reported that when patients were not provided with a prescription for antimicrobials, patients left unsatisfied and some providers believed they would likely go to a walk-in clinic to obtain an antimicrobial for their infection.

“*Patient expectations and their previous experiences with antibiotics for certain conditions*. *For example*, *bronchitis a lot of them say they always get antimicrobials previously and that may have been the thinking back then so that is one of the biggest factors in terms of trying to manage those expectations and the need for antibiotics*.*”* (Site 1 Family Physician 3)

“*Patient demand—my biggest one is patients*. *It is getting better*, *but the reality is patients push [saying] ‘my other doctor used to give it to me all the time and whenever I take it I feel better’*.*”* (Site 2 Family Physician 1)

“*I am pretty sure she went somewhere else and got a prescription but at least I felt like I had pushed for the guidelines but that does affect you when the patients are really pushing for it*.*”* (Site 1 Nurse Practitioner)

#### 2. Being influenced by situational factors

Participants noted the likelihood of prescribing antimicrobials was influenced by the day of the week with more prescriptions being provided on Fridays (usually with the instructions to delay the prescription until lab results obtained). Also affecting prescribing was whether the patient had already been seen elsewhere and was not getting better.

“*The day of the week in this office setting probably affect it for example like a Friday before a long weekend you know we might be more open to giving like a delayed prescription or a prescription for antimicrobial if there can’t be a good follow-up*.*”* (Site 3 Family Physician 6)

*A lot of the time patients have been seen in walk-in clinics and already have been given something and then it is not working*, *and they come to see me for something else or something better*.*”* (Site 3 Family Physician 1)

#### 3. Experiencing diagnostic uncertainty based on clinical factors

Several prescribers mentioned the clinical presentation and the severity and length of the symptoms as influencing whether they prescribed an antimicrobial or not. The following narrative excerpts illustrate this theme.

“*Severity of symptoms so the combination of severity and lasting a long time*.*”* (Site 1 Family Physician 7)

“*The patient’s clinical presentation with it*. *If watching and waiting is going to be harmful or waiting to get a definitive culture is going to be harmful*.*”* (Site 2 Family Physician 2)

“*Another factor is (the) patient*. *You have to look at the patient*, *the overall picture of the patient like comorbidities and that sort of thing so age and presence of other medical conditions*.*”* (Site 2 Family Physician 4)

#### 4. Believing that providers were already prescribing judiciously with little room for improvement

This theme reflects how most participants viewed the ASP to be valuable and relevant, however several noted that it was not *“a foreign concept”* and served to *“reinforce what we already knew and were doing”* and *“strengthen their conviction around certain practice patterns”*. Referred to as *“preaching to the converted”* by one family doctor, the majority of study participants described themselves as being *“judicious about antibiotic use”* and believed there was minimal room for improvement or need to change practice.

“*The program reiterated and reinforced things for some of the conditions where maybe I would have been more inclined to use antimicrobials for bronchitis and after the program sort of really minimized any use of antibiotics for that*. *This wasn’t a foreign concept*.*”* (Site 1 Family Physician 4)

“*It confirmed a lot of what I was doing already… I was already generally not prescribing so they were nice reminders*.*”* (Site 2 Family Physician 4)

“*My understanding was good*, *I was already using my antibiotics pretty judiciously*, *so I didn’t think there would be much room for me to improve*.*”* (Site 3 Family Physician 2)

#### 5. Having time pressures affecting prescribing and learning about antimicrobial stewardship

Study participants identified challenges finding time to complete the ASP educational components, accessing the PC-ASP components during clinic, and using the communication scripts with patients. Some prescribers also described having to take more time to provide education and convince patients that they have a viral infection and don’t require an antimicrobial compared to just writing a prescription.

“*Making the time to read the modules*. *There are always competing demands and you lose energy in one project or another*, *this should really be embedded in our day-to-day practice… We’re busy…”* (Site 1 Family Physician 6)

“*It might be on the desktop but if they don’t know exactly where to find it and they only have 15 minutes and running behind they will go with what is at the top of their head*.*”* (Pharmacist did not want site to be identified)

“*I am more likely to prescribe an antibiotic if I feel that I am in a rush because having a discussion takes more time*. *Similarly*, *if I am working a walk-in clinic and I can see that there are many people waiting to be seen I might be more likely to you know take the easy way out*.*”* (Site 3 Family Physician 3)

## Discussion

This qualitative study of the perceptions of Canadian primary care providers regarding the usefulness of, and potential barriers to, multi-faceted antimicrobial stewardship in primary care identified factors relevant to community ASP implementation efforts. Some factors appeared to facilitate participation in ASP activities while others represented barriers identified by these providers.

Other qualitative studies of antibiotic prescribing in primary care have focused on either a limited set of interventions [21,22] or a limited set of factors to understand influences on the uptake of ASP activities in primary care. [39] A European internet-based educational program to promote the use of C-reactive protein testing and prescribing-specific communication skills found country-specific differences in communication style and health systems that limited applicability. [36] An American study of pediatricians who received an educational intervention plus audit and feedback of prescribing practices reported ‘deep skepticism’ about the validity of the audit data. [21] A French study of the use of a specific antibiotic prescription with educational messaging for patients found physicians viewed this as excessive. [22] These findings identify potential barriers to primary care ASP efforts, some of which may be specific to certain interventions.

Other studies point to the importance of patient and system factors. The willingness of Australian general practitioners to use delayed antibiotic prescriptions was influenced by their knowledge of the intervention and interpersonal skills, but also by expectations for antibiotics by patients and concern about misuse of delayed prescriptions. [40] Patient pressure or expectations for antibiotics has been consistently commented on by providers in various studies as a barrier to appropriate antibiotic use, [41,42] and was also a concern of providers in the current study. Reimbursement issues, the need for quality control and impact on work flows were other factors identified in a European study affecting the adoption of a point of care c-reactive protein test in primary care clinics to limit antibiotic use in lower respiratory infections. [43]

The current study identified additional factors with the potential to influence the incorporation of multi-faceted ASP activities into clinical practice. These included having a local champion to handle the organizational issues and ongoing communication, prompts during clinical activities to remind them to think about their ASP and options, having enough time to learn about new ASP information, and ongoing audit and feedback regarding antimicrobial prescribing practices.

Guidelines for institutional ASP programs recommend a dedicated stewardship team with institution-provided support for the personnel and infrastructure to allow for ongoing audit and feedback of prescribing practices as a critical success factor. [13] The views expressed by primary care providers in our study suggest parallel structures and processes may be needed in the community. In Scotland, a model utilizing a primary care antimicrobial management team was utilized as part of a national initiative to reduce broad spectrum antimicrobial prescribing in a large region. [44] In the current study, a local team of a physician, pharmacist and administrative staff acted as site champions to promote the uptake of ASP information and resources.

This involvement of a designated ‘champion’ team was generally viewed as positive and necessary for clinic ASP efforts by these providers. Previous studies have pointed to local champions as a “critical” support for implementing change in primary care.[25] A study of antibiotic prescribing in an American pediatric network of practices reported a clinic’s readiness to change and having active processes for supporting change as additional factors associated with prescribing changes.[39] The current study provides support for the importance of stewardship champions in facilitating local clinic processes that promote ASP efforts, and possibly influencing the readiness of primary care groups to adopt stewardship practices.

Audit and feedback of antimicrobial prescribing is a recommended activity for community ASPs [15]. It has been found to be effective in reducing antimicrobial use, [27, 28] as long as the process is ongoing. [45] The audit and feedback component of the current ASP program was reported by these providers to be useful and relevant. This is in contrast to a study of pediatricians who reported being skeptical of the data on which their prescribing audit was based. [21] Our study of primary care providers did not elicit concerns about the credibility of the data in the feedback reports. This difference may in part be explained by differing report formats in the two studies, and because the current study used clinic level prescribing compared to individual prescriber data in the pediatric study. [45]

Some providers in our study felt they used antibiotics judiciously already, a view that would not be challenged with clinic level reports. The belief by some physicians that they are ‘good’ antimicrobial prescribers was also found in studies of Irish general practitioners [46] and American pediatricians. [21] While such beliefs may serve as a barrier to community ASP efforts, individual data may potentially affect engagement of providers unless the credibility of the reports can be assured. [21] Additionally, while an American study of primary care doctors found an important 5% absolute reduction in antibiotic prescribing with individualized peer comparison [47], a large European study found no effect [48]. Standardized coding of the clinical indication for prescribing an antibiotic has been suggested as a way to improve audit data quality in primary care. [17]

Other potential barriers to ASP activities in primary care identified in the current study and others included time constraints to learning about ASP content, [22,36] the time needed during clinical encounters to explain decisions not to prescribe an antimicrobials, [40,43] patient dissatisfaction with not being prescribed antibiotics, [40,46,49] and uncertainty brought about by clinical or situational factors such as the day of the week being before a weekend. [40]

Two specific strategies had been included in the PC-ASP to assist providers in addressing patient expectations and concerns about not receiving antimicrobials (delayed prescriptions [35] and condition-specific communication points. [33,34] However, this did not appear to be recognized by participants as none made comments linking the two. This may have been because an acknowledgment of the experience of patient expectations was not part of the education materials, and an explicit link was not made to the provided clinical strategies as ways to address patient expectation. An implication for ASP programs is that educational components should address the experience of perceived patient pressure that providers encounter, and link this explicitly to the strategies that help clinicians to recognize and address patient concerns.

Limitations to the current study are it was a short-term study conducted in a large urban center, with most clinics involved in teaching of family physician trainees. General practitioner trainers have been noted to prescribe fewer antimicrobials in one study. [50] Participation in the qualitative interviews was voluntary. These factors may have resulted in a less diverse spectrum of clinical providers than is present in the wider community. Nonetheless, the viewpoints of participant group converged on common themes supported by other studies of antimicrobial prescribing. This study adds the perspectives of Canadian primary care providers regarding multi-faceted ASP interventions, and the factors they see as facilitators and barriers to their implementation in the community.

## Conclusion

This qualitative study of primary care providers confirmed some findings of previous research examining barriers and facilitators of single interventions to promote the optimal use of antimicrobials as also likely relevant in multi-faceted community ASP programming. Addressing time constraints facing busy clinical providers and explicitly identifying communication strategies and delayed prescribing opportunities to address provider concerns about patient expectation for antimicrobials are especially important. In addition, a local person or team responsible for ASP programming and providing audit and feedback was viewed as necessary to the success of community-based ASP. Including these aspects as part of ASP programming in primary care may require additional resources to support primary care clinics in adopting ASP practices and address antibiotic overuse in the community.
